# Salicylic Acid Spray Delays Sand Pear Fruit Senescence during Room Temperature Shelf Life by Regulating Antioxidant Capacity and Senescence-Related Genes

**DOI:** 10.3390/plants13060848

**Published:** 2024-03-15

**Authors:** Huiying Wang, Yawei Li, Misganaw Wassie, Liyue Huo, Haiyan Shi

**Affiliations:** 1 College of Horticulture, Hebei Agricultural University, Baoding 071001, China; why0108483x@163.com (H.W.); liyawei589@163.com (Y.L.); hly202138@163.com (L.H.); 2 Xishuangbanna Tropical Botanical Garden, Chinese Academy of Sciences, Kunming 666300, China; misgie2018@wbgcas.cn

**Keywords:** ‘Whangkeumbae’ pear, salicylic acid, antioxidant capacity, senescence, gene regulation

## Abstract

‘Whangkeumbae’ (*Pyrus pyrifolia*) is a variety of sand pear fruit well-known for its smooth surface and good taste. However, the fruit quality is adversely affected by postharvest ethylene production. Therefore, improving postharvest shelf life by regulating fruit senescence is critical to promoting the ‘Whangkeumbae’ fruit industry. Here, we investigated the effect of salicylic acid (SA) spray on fruit senescence in sand pears during room temperature shelf life. Exogenous SA reduced polyphenol oxidase (PPO) activity and malondialdehyde (MDA) content during room temperature shelf life. Additionally, SA effectively maintained the fruit skin coloration and increased the activity of antioxidant enzymes, such as superoxide dismutase (SOD), peroxidase (POD), catalase (CAT), and ascorbate peroxidase (APX). SA treatment inhibited *PpPPO1* expression and upregulated *PpSOD1*, *PpAPX6*, and *PpGST2* expression. Furthermore, SA application downregulated the expression of *PpACO2*, *PpEIN3a*, *PpNCED1*, and *PpAOC2*, while upregulating *PpNPR-1*, *PpTAR2*, and *PpCOMT1* during room temperature shelf life. SA treatment also influenced cell wall metabolism and modification genes by inhibiting *PpPG1*, *PpPME2*, and *PpCEL3* and inducing *PpPGIP1* expression. Additionally, SA treatment affected sugar and acid metabolism genes and increased the expression of *PpSPS1*, *PpSUS1*, *PpSOT1*, *PpTMT4*, *PpSWEET15*, and *PpcyNAD-MDH,* but suppressed the expression of *PpcyNADP-ME*. The Pearson correlation analysis indicated that PPO activity and MDA content were positively correlated with the expression of *PpPPO1*, *PpACO2*, *PpEIN3a*, *PpNCED1*, *PpAOC2*, *PpPG1*, *PpPME2*, *PpCEL3*, and *PpcyNDA-MDH*. Conversely, these factors were negatively associated with the activities of SOD, POD, CAT, and APX, as well as the expression levels of *PpSOD1*, *PpPOD1*, *PpCAT1*, *PpAPX6*, *PpGST2*, *PpNPR-1*, *PpTAR2*, *PpCOMT1*, *PpPGIP1*, *PpSPS1*, *PpSUS1*, *PpSOT1*, *PpTMT4*, *PpSWEET15*, and *PpcyNAD-MDH*. Our results reveal that exogenous SA could delay fruit senescence in sand pear fruit by regulating various biochemical and molecular mechanisms and can be used to effectively extend fruit shelf life during room temperature storage. However, further research is necessary to determine whether the fruits sprayed with SA are suitable for direct human consumption.

## 1. Introduction

‘Whangkeumbae’ (*Pyrus pyrifolia* Nakai) is a well-known sand pear fruit with a tender, juicy, sweet, and sour taste [1]. However, sand pear fruit can be spoiled when stored at room temperature for an extended period, thus affecting its commercial and economic value [2,3]. Therefore, delaying sand pear fruit senescence and extending its shelf life is essential to reducing economic losses.

During fruit senescence, several physiological changes could occur, including the over-accumulation of reactive oxygen species (ROS), membrane lipid peroxidation, and cell membrane damage [4,5,6,7]. In plant cells, ROS, including superoxide (O_2_^•−^), singlet oxygen (^1^O_2_), (H_2_O_2_), and the hydroxyl radical (^•^OH) are produced in different cellular compartments and induce oxidative stress, thus leading to cell damage and death [8,9]. Accumulating evidence shows that fruit senescence is primarily facilitated by increased ROS accumulation [10,11,12]. Meanwhile, plants develop sophisticated enzymatic and non-enzymatic antioxidant defense systems to eliminate over-accumulated ROS [13,14]. Previous studies have shown that antioxidant enzymes, such as superoxide dismutase (SOD), peroxidase (POD), catalase (CAT), and ascorbate peroxidase (APX), could play a significant role in delaying postharvest fruit senescence by reducing ROS accumulation [8,15,16]. The increase in antioxidant enzyme activity during the senescence of peach fruit is associated with an increase in the expression level of antioxidant related genes [17]. Glutathione transferases (GSTs) protect tissues against oxidative stress or toxic products produced during exogenous metabolic processes [18]. Additionally, *PpGST* is involved in the fruit ripening and senescence of pears [19].

Ethylene is a critical plant hormone in plant growth, development, and stress responses [20]. It is a primary hormone responsible for rapid fruit ripening and senescence, affecting postharvest product quality, especially in climacteric fruits [21,22]. Therefore, controlling ethylene production is critical to extending fruit shelf life, and various strategies have been developed to inhibit ethylene biosynthesis and signal transduction [23,24]. For instance, exogenous chemical treatment has been used as a practical approach to extend fruit shelf life. During ethylene biosynthesis, 1-aminocyclopropane-1-carboxylic acid (ACC) synthase (ACS) catalyzes *S*-adenosyl-*L*-methionine (SAM) to ACC. Subsequently, ACC oxidase (ACO) converts ACC to ethylene [25]. Previous reports have shown that fruit ripening is delayed by regulating the expression of the two key ethylene biosynthesis genes, *ACS* and *ACO* [26]. EIN3 is an important transcription factor (TF) in the ethylene signaling pathway. Ethylene is sensed by its receptors [27]. The ethylene signaling pathway is initiated by the EIN3/EIL (ethylene-insensitive 3/EIN3-like) TF, which activates the ethylene response factor (ERF) TF, and then regulates the expression of ethylene-responsive genes [27].

Salicylic acid (SA) is a phenolic phytohormone that regulates plant growth, development, defense against pathogens, and abiotic stress responses [28,29,30]. SA is a natural and safe chemical preservative with a profound effect on delaying fruit senescence [31,32,33]. Accumulating evidence showed that the application of SA could effectively delay fruit senescence in various fruits, such as cherry [34,35,36], citrus [37], guava [38], banana [39], longan [40,41], blueberry [42], grape [43], apple [44,45], apricot [46], strawberry [47], pomegranate [48], and peach [49]. For instance, SA treatment effectively inhibited the browning of the pear pulp tissue, maintained good postharvest quality, and prolonged the fruit storage period [50]. Additionally, SA remarkably maintained the firmness of peach fruit and reduced membrane lipid peroxidation, thereby extending its shelf life during cold storage [51]. NPR1 is a high-affinity SA-binding protein involved in SA-induced immunity. NPR1 has the ability to promote the expression of SA-induced defense genes.

Under cold and room temperature storage conditions, SA delayed the softening of apricot fruit by enhancing the activities of antioxidant enzymes, thereby extending its shelf life [52,53]. Our recent study showed that SA could delay fruit senescence in sand pears by regulating *PpEIN3a* and playing an antagonistic role with ethylene, auxin, and glucose [54]. However, the detailed mechanism of how exogenous SA regulates fruit senescence during extended room temperature storage remains poorly understood, especially in sand pears.

The primary components of the fruit cell wall are pectin, cellulose, and hemicellulose [55]. Fruit softening is directly related to the activity of cell wall-modifying enzymes, including polygalacturonase (PG), pectin methyl esterase (PME), and cellulase (Cx). The increased activity of these enzymes accelerates fruit softening [56]. Polygalacturonase inhibitors (PGIPs) play a crucial role in plant defense against fungal pathogens by inhibiting the pectin-depolymerizing activity of polygalacturonase [57,58]. Soluble sugars are essential for the growth, development, and quality of pear fruit [59], and their ratio significantly influences the flavor [60]. The accumulation of sugars in higher plants involves the complex regulation of metabolic enzymes under the control of plant hormones [61].

In this study, we investigated the regulatory effect of exogenous SA spray on fruit senescence in sand pears during room temperature shelf life. The results demonstrated that exogenous SA spray could mitigate the deleterious effects of the room temperature shelf life and delay fruit senescence by modulating antioxidant systems, plant hormone synthesis and metabolism, cell wall metabolism and modification, and sugar-acid metabolism.

## 2. Results

### 2.1. Effects of SA Treatment on Pear Fruit Coloration during Room Temperature Shelf Life

**Figure 1:**
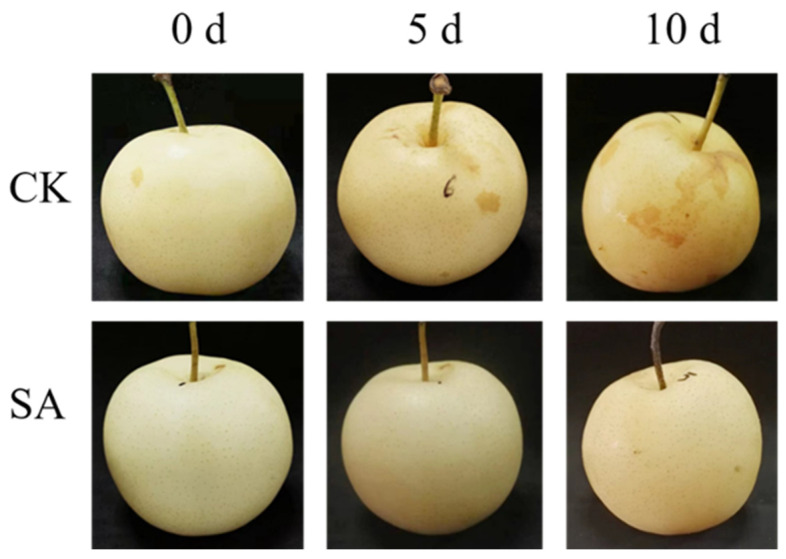
Effects of exogenous SA spray on pear fruit skin coloration during room temperature shelf life.

First, the effects of exogenous SA spray on postharvest fruit coloration were investigated. Room temperature shelf life for 5 days and 10 days significantly affected fruit coloration. For instance, the skin color of pear fruits changed from yellow-green to yellow at 5 days and yellowish-brown at 10 days compared to the SA-untreated fruits. Interestingly, exogenous SA application remarkably maintained skin coloration, and fruits treated with SA for 5 days and 10 days showed comparable fruit coloration with the 0-day fruits (Figure 1). For instance, pear fruits treated with SA for 5 days and 10 days exhibited yellow-green and yellow fruit coloration, respectively, suggesting that the exogenous application of SA could maintain fruit coloration in pear during room temperature shelf life.

### 2.2. Effects of SA Treatment on PPO Activity and MDA Content in Pear Fruit during Shelf Life at Room Temperature

**Figure 2:**
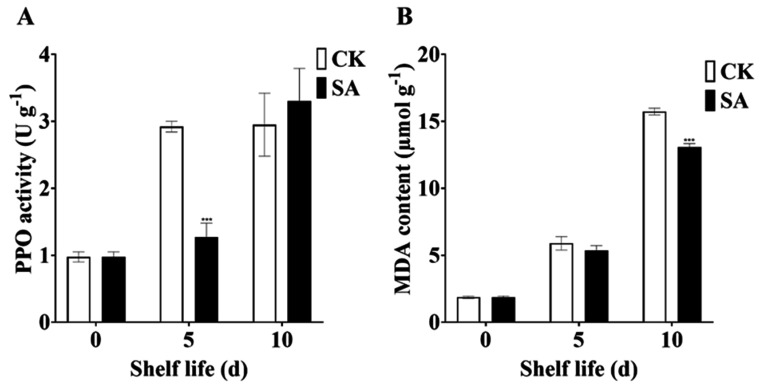
Exogenous SA regulates PPO activity and MDA content in sand pear fruits during shelf life at room temperature. (**A**) PPO activity. (**B**) MDA content. The data in the figure are given as mean (n = 3). The bars represent the standard error. Asterisk indicates statistically significant differences at *** *p* < 0.001 based on Duncan’s test.

Room temperature shelf life significantly increased the PPO activity in pear fruits, especially at 5 days. Conversely, SA treatment for 5 days significantly reduced the PPO activity compared to the SA-untreated fruits, while the SA spray for 10 days had no significant effect on PPO activity during the shelf life at room temperature (Figure 2A).

We further measured the MDA content to check the effects of exogenous SA on maintaining membrane stability in pear fruit. Room temperature shelf life for 5 days increased the MDA content of pear fruits, and the increment trend was further elevated at 10 days. Interestingly, pear fruits treated with exogenous SA for 10 days showed significantly lower MDA content compared to the non-SA-treated fruits during shelf life at room temperature (Figure 2B).

### 2.3. Exogenous SA Application Regulates Antioxidant Enzymes in Sand Pear Fruits during Shelf Life at Room Temperature

**Figure 3:**
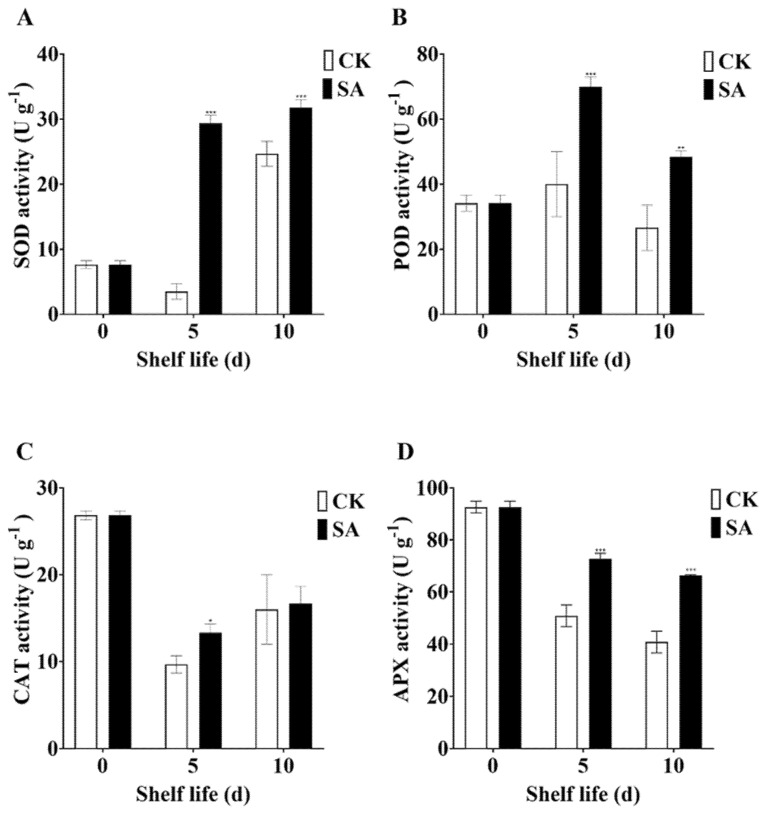
Effects of SA treatment on the antioxidant enzyme activity in pear fruit during shelf life at room temperature. (**A**) SOD activity. (**B**) POD activity. (**C**) CAT activity. (**D**) APX activity. The data in the figure are given as the mean (n = 3). The bars represent the standard error. The asterisk indicates statistically significant differences at * *p* < 0.05 or ** *p* < 0.01 or *** *p* < 0.001 based on Duncan’s test.

Postharvest SA treatments can modulate ROS accumulation by regulating the activity of antioxidant enzymes [7]. Thus, to understand how SA regulates ROS accumulation in pear fruits during shelf life at room temperature, we measured the activity of several antioxidant enzymes, including SOD, POD, CAT, and APX. Compared to the SA-untreated fruits, SOD activity was reduced at 5 days, but increased at a 10-day room temperature shelf life. Exogenous SA spray increased the SOD activity by 8.4-fold during a 5-day room temperature shelf life (Figure 3A). The SOD activity was further elevated at 10-day SA treatment compared to the 5-day SA treatment and the SA-untreated fruits. Compared to the SA-untreated fruits, room temperature shelf life for 5 days increased POD activity in pear fruits, while extending the shelf life to 10 days reduced POD activity in SA-untreated and SA-treated fruits. Interestingly, POD activity was increased by SA application during shelf life at room temperature (Figure 3B). In addition, room temperature shelf life and SA treatment reduced CAT activity compared to SA-untreated. But, pear fruits treated with SA for 5 days exhibited significantly higher CAT activity than the SA-untreated fruits during shelf life at room temperature (Figure 3C). SA treatment had no notable effect on CAT activity at a 10 room temperature shelf life. On the other hand, room temperature storage and SA treatment dramatically reduced the APX activity compared to the SA-untreated control, and the effect was more pronounced with increasing shelf life. Interestingly, pear fruits treated with SA for 5 days and 10 days showed significantly higher APX activity than the SA-untreated fruits during shelf life at room temperature (Figure 3D).

### 2.4. Analysis of Expression Levels of Genes Related to Antioxidant Enzyme Activity during Pear Fruit Senescence

**Figure 4:**
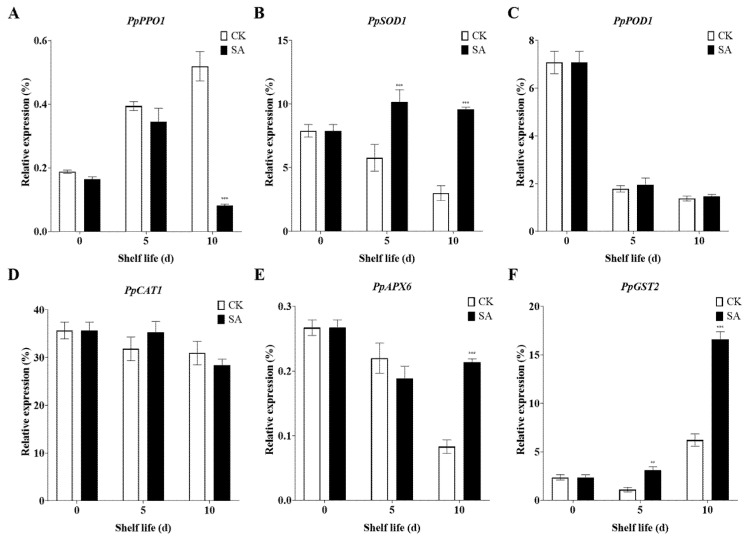
Effects of SA treatment on the expression of antioxidant enzyme genes in sand pears during shelf life at room temperature. (**A**) *PpPPO1*. (**B**) *PpSOD1*. (**C**) *PpPOD1*. (**D**) *PpCAT1*. (**E**) *PpAPX6*. (**F**) *PpGST2*. The data in the figure are given as the mean (n = 3). The bars represent the standard error. The asterisk indicates statistically significant differences ** *p* < 0.01 or *** *p* < 0.001 based on Duncan’s test.

To elucidate the molecular mechanisms of how exogenous SA regulates antioxidant enzymes in pear fruit, we further measured the expression of related genes, including *PpPPO1*, *PpSOD1*, *PpPOD1*, *PpCAT1*, *PpAPX6*, and *PpGST2*. The expression of *PpPPO1* was significantly induced during shelf life at room temperature. However, exogenous SA inhibited the expression of *PpPPO1* during shelf life at room temperature, especially 10 days after SA treatment (Figure 4A). The expression level of *PpSOD1* significantly decreased during shelf life at room temperature in the SA-untreated fruits. While exogenous SA significantly induced the expression of *PpSOD1* during shelf life at room temperature, with a peak expression at 5 days after SA treatment (Figure 4B). Room temperature shelf life dramatically reduced the expression of *PpPOD1* in pear fruit. However, exogenous SA did not significantly affect the expression of *PpPOD1* under room temperature shelf life (Figure 4C). Room temperature shelf life slightly reduced the expression of *PpCAT1* in the SA-untreated fruits. In addition, SA spray had no significant effect on the expression of *PpCAT1* compared to the non-SA-treated fruits during shelf life at room temperature (Figure 4D). Moreover, room temperature shelf life decreased the expression of *PpAPX6*. Although SA treatment for 5 days reduced the expression of *PpAPX6*, extending SA treatment for 10 days significantly increased *PpAPX6* expression compared to the non-SA-treated fruits (Figure 4E). In addition, *PpGST2* showed reduced expression at 5 days in the SA-untreated fruits, but SA spray induced its expression at the same storage time. While *PpGST2* expression was activated at 10 days of room temperature shelf life, SA treatment significantly increased its expression compared to the non-SA-treated fruits (Figure 4F).

### 2.5. SA Regulates the Expression of Plant Hormone Synthesis and Metabolism Genes during Shelf Life at Room Temperature

**Figure 5:**
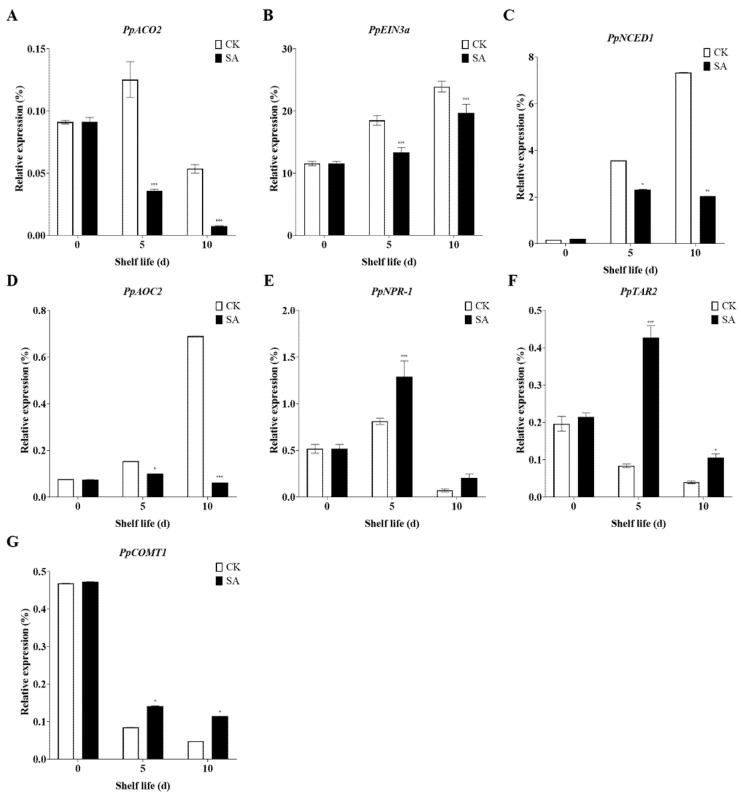
SA regulates the expression of plant hormone synthesis and metabolism genes during shelf life at room temperature. (**A**) *PpACO2*. (**B**) *PpEIN3a*. (**C**) *PpNCED1*. (**D**) *PpAOC2*. (**E**) *PpNPR-1*. (**F**) *PpTAR2*. (**G**) *PpCOMT1*. The data in the figure are given as the mean (n = 3). The bars represent the standard error. The asterisk indicates statistically significant differences at * *p* < 0.05 or ** *p* < 0.01 or *** *p* < 0.001 based on Duncan’s test.

Different plant hormones form complex networks and regulate the postharvest quality of fruits through their synergistic or antagonistic interactions [23]. Thus, we investigated the effects of SA spraying on the expression of plant hormone synthesis and metabolism genes, such as *PpACO2*, a gene related to ethylene synthesis [62], and *PpEIN3a*, a gene involved in the ethylene signal transduction pathway [54]. Pear fruits placed at room temperature for 5 days and 10 days exhibited a lower expression of *PpACO2* compared to the SA-untreated fruits (Figure 5A). In addition, pear fruits placed at room temperature for 5 days and 10 days showed a higher expression of *PpEIN3a* than the SA-untreated fruits. Interestingly, SA application significantly reduced the expression of *PpEIN3a* at 5 days and 10 days during shelf life at room temperature compared to the non-SA-treated fruits (Figure 5B). Additionally, genes involved in plant hormone synthesis and metabolism, such as *NCED* [63], *AOC* [64], *PpNPR-1* [65], *TAR* [66], and *COMT* [67], play crucial roles in fruit ripening and senescence. RT-qPCR analysis of the related genes in sand pears showed that SA treatment decreased the expression of *PpNCED1* and *PpAOC2* (Figure 5C,D), while increasing *PpNPR-1* expression (Figure 5E). The expression levels of *PpTAR2* and *PpCOMT1* decreased during storage life, but SA treatment upregulated their expression (Figure 5F,G).

### 2.6. Exogenous SA Treatment Regulates Cell Wall Metabolism and Modification-Related Genes Expression

**Figure 6:**
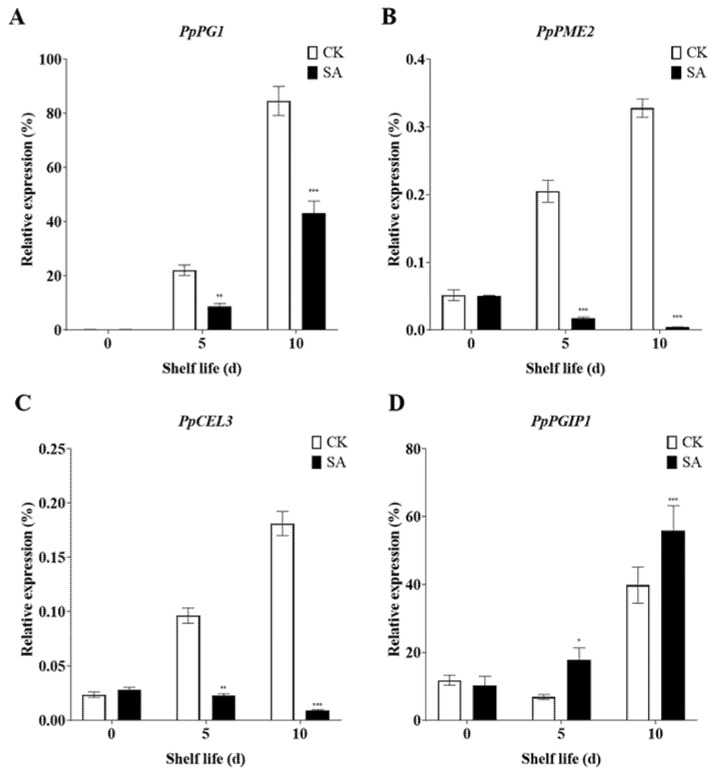
Exogenous SA regulates the expression levels of cell wall metabolism and modification-related genes. (**A**) *PpPG1*. (**B**) *PpPME2*. (**C**) *PpPCEL3*. (**D**) *PpPGIP1*. The data in the figure are given as the mean (n = 3). The bars represent the standard error. The asterisk indicates statistically significant differences at * *p* < 0.05 or ** *p* < 0.01 or *** *p* < 0.001 based on Duncan’s test.

The change in fruit firmness can affect fruit quality and shelf life, and cell wall metabolism-related genes such as *PG*, *PME*, and *CEL* play a critical role in fruit firmness [68]. Thus, we measured the expression of the cell wall metabolism-related genes, such as *PpPG1*, *PpPME2*, and *PpCEL3*. The results indicate that the expression of these genes increased during shelf life, but SA treatment significantly decreased the expression levels of *PpPG1*, *PpPME2*, and *PpCEL3* (Figure 6A–C). The expression level of *PGIP* is closely related to fruit firmness [69]. Interestingly, *PpPGIP1* showed reduced expression at 5 days in SA-untreated fruits, but exogenous application of SA induced its expression at the same shelf-life time (Figure 6D).

### 2.7. SA Regulates the Expression of Genes Related to Sugar and Acid Metabolism during Room Temperature Shelf Life

**Figure 7:**
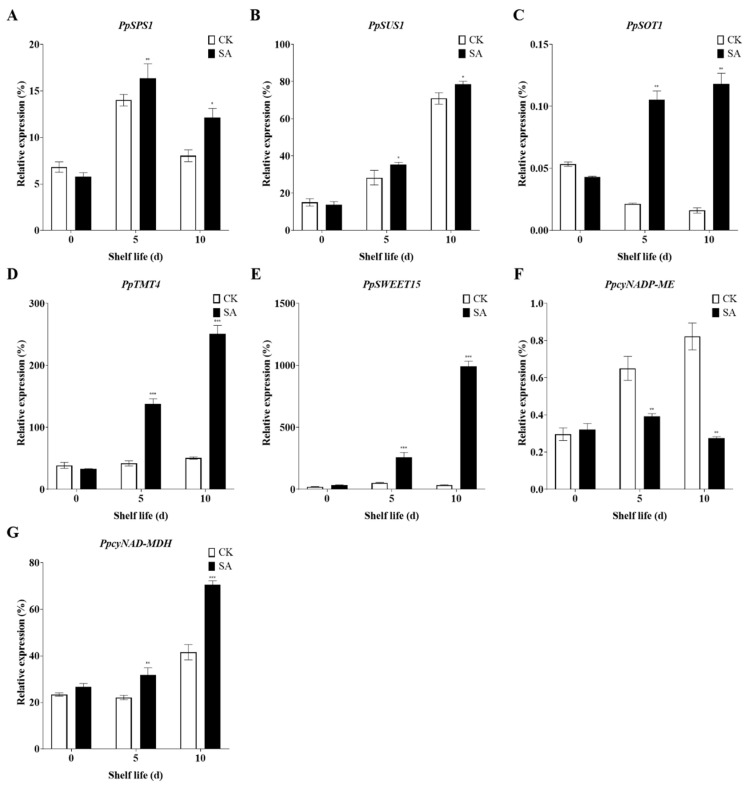
SA regulates the expression of genes related to sugar and acid metabolism during shelf life at room temperature. (**A**) *PpSPS1*. (**B**) *PpSUS1*. (**C**) *PpSOT1*. (**D**) *PpTMT4*. (**E**) *PpSWEET15*. (**F**) *PpcyNADP-ME*. (**G**) *PpcyNAD-MDH*. The data in the figure are given the mean (n = 3). The bars represent the standard error. The asterisk indicates statistically significant differences at * *p* < 0.05 or ** *p* < 0.01 or *** *p* < 0.001 based on Duncan’s test.

The content of soluble sugars and organic acids is crucial to the quality of pear fruit. Genes associated with sugar and acid metabolism, namely *SPS* [70], *SUS* [71], *SOT* [72], *TMT* [73], *SWEET* [74], *cyNADP-ME,* and *cyNAD-MDH* [75], play pivotal roles in determining the levels of these compounds in pear fruit. We further investigated the effect of SA on the expression of these genes in pear fruit during shelf life. The expression of sucrose synthesis-related genes, *PpSPS1* and *PpSUS1,* increased during the shelf life. SA treatment significantly upregulated the expression of *PpSPS1* and *PpSUS1*, thus promoting sucrose synthesis (Figure 7A,B). The sugar transporter-related gene *PpSOT1* decreased during shelf life (Figure 7C), while *PpTMT4* and *PpSWEET15* showed no significant changes (Figure 7D,E). SA treatment significantly upregulated the expression of *PpSOT1*, *PpTMT4,* and *PpSWEET15* (Figure 7C–E). The expression levels of malic acid metabolism-related genes, *PpcyNADP-ME* and *PpcyNAD-MDH,* were upregulated during shelf life (Figure 7F,G). After treatment with SA, the expression levels of these two genes exhibited opposite trends. Specifically, the expression of *PpcyNADP-ME* was down-regulated (Figure 7F), whereas the expression of *PpcyNAD-MDH* was upregulated, thereby increasing the malic acid content (Figure 7G).

### 2.8. Pearson Correlation Analysis of Senescence-Related Indexes

**Figure 8:**
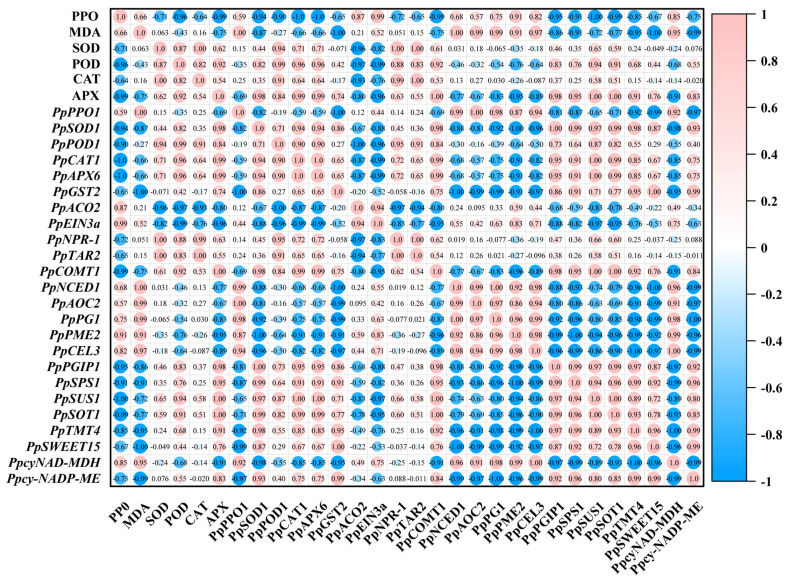
Correlation analysis of different indexes for SA treatment during shelf life at room temperature. Red color indicates strong positive correlations, while blue color represents strong negative correlations.

**Figure 9:**
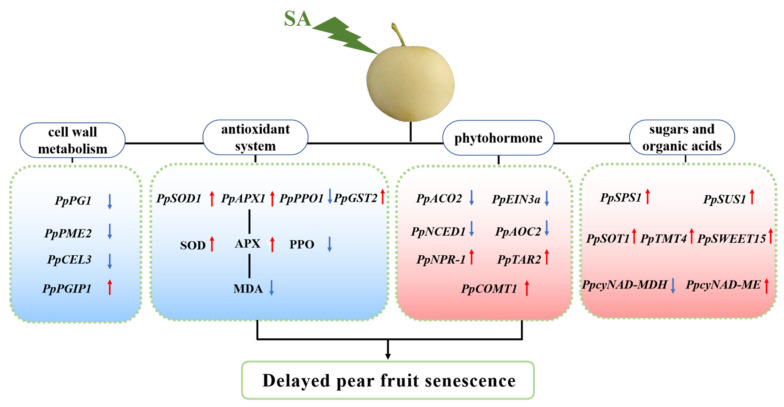
A hypothetical working model illustrating the role of SA in delaying pear fruit senescence during shelf life at room temperature. SA increases antioxidant activity, reducing ROS accumulation, thereby delaying fruit senescence. Red arrows represent an up-regulation, and blue arrows represent a down-regulation.

Furthermore, Pearson correlation analysis was conducted to evaluate the relationships among the measured parameters. As shown in Figure 8, a positive correlation was observed between PPO activity and MDA content. In contrast, a negative correlation was observed between PPO activity and SOD, POD, CAT, and APX activities. Notably, significant correlations existed among genes related to hormone synthesis and metabolism. The salicylic acid signal transduction gene, *PpNPR-1*, was positively correlated with the auxin synthesis-related gene, *PpTAR2*, and the melatonin synthesis-related gene, *PpCOMT1*. Conversely, *PpNPR-1* was negatively associated with ethylene synthesis and signal transduction genes (*PpACO2* and *PpEIN3a*), the abscisic acid synthesis gene *PpNCED1*, and the jasmonic acid synthesis gene *PpAOC2*. Furthermore, cell wall metabolism and modification genes were strongly correlated. *PpPG1*, *PpPME2*, and *PpCEL3* were positively correlated, whereas a negative correlation was observed with *PpPGIP1*. Genes related to glucose and acid metabolism, such as *PpSPS1*, *PpSUS1*, *PpSOT1*, *PpTMT4*, *PpSWEET15*, and *PpcyNAD-MDH*, demonstrated a strong positive correlation while showing a negative correlation with *PpcyNADP-ME*. A strong correlation was evident between the various metabolic pathways. The activities of antioxidant enzymes (SOD, POD, CAT, and APX) and the expression of related genes were positively correlated with *PpNPR-1*, *PpTAR2*, and *PpCOMT1,* yet negatively correlated with *PpACO2*, *PpEIN3a*, *PpNCED1*, and *PpAOC2*. The activities of antioxidant enzymes and the expression of related genes also exhibited a negative correlation with cell wall metabolism and modification-related genes (*PpPG1*, *PpPME2*, and *PpCEL3*) while maintaining a positive correlation with the sugar and acid metabolism-related genes (Figure 8). Overall, SA treatment could delay pear fruit senescence and maintain fruit quality by coordinately regulating various metabolic pathways during room temperature shelf life (Figure 9).

## 3. Discussion

Rapid postharvest fruit senescence is a severe problem that limits fruit quality. Therefore, maintaining fruit quality by increasing postharvest fruit shelf life is a significant concern in the fruit industry. Given this, applying different chemicals and plant hormones have been used as an effective strategy to enhance the shelf life of fruits. For instance, the postharvest application of melatonin and 1-methylcyclopropene remarkably improved the shelf life of ‘Hayward’ kiwifruit during storage [76]. Additionally, the exogenous application of SA has improved the postharvest shelf life and quality of ‘Jinshayou’ pummelo fruits [7].

It is well-established that storage temperature is a critical factor in determining postharvest fruit quality [77,78]. In this study, we investigated the effects of exogenous SA treatment on the color change in pear fruits during shelf life at room temperature. The storage duration had a noticeable impact on fruit coloration on the control fruits, shifting from yellow-green to yellowish-brown over time. However, the application of exogenous SA effectively maintained fruit coloration, as evidenced by the comparable coloration of SA-treated fruits to the control group. Previous studies have shown that elevated temperatures could affect skin coloration in various fruits [78,79,80]. The change in skin coloration in sand pear fruits during shelf life at room temperature indicates the process of fruit senescence caused by elevated temperature. Interestingly, the exogenous application of SA reduces the negative consequences during shelf life at room temperature. Pear fruits treated with SA showed green to yellow skin coloration, indicating that SA could effectively delay fruit senescence in sand pears during shelf life at room temperature.

Enzymatic browning is a central reaction that negatively affects fruit color, taste, and quality [81,82]. PPO is a primary enzyme that induces fruit browning by oxidation of phenolic substances [83,84]. In this study, postharvest room temperature shelf life increased the activity of PPO in sand pear fruits, which ultimately caused fruit browning. Interestingly, exogenous SA significantly reduced the PPO activity in sand pears during shelf life at room temperature, and the fruits maintained yellow coloration. Similarly, SA treatment decreased PPO activity and reduced internal browning symptoms in pear fruit [85]. Additionally, the expression level of *PpPPO1* in the SA-treated group was significantly lower than that in the control at 10 days, similar to the expression pattern of *PbPPO4* in ‘Yali’ pears [86].

A higher ROS accumulation contributes to oxidative stress and membrane lipid peroxidation in plant cells, adversely affecting membrane integrity [87]. The level of cell membrane damage has been measured by the change in MDA content [87,88]. In this study, room temperature dramatically increased MDA content in sand pear fruits, which could be associated with lower antioxidant enzyme activity and higher ROS accumulation. However, SA spray remarkably reduced the MDA content of sand pear fruits placed at room temperature, suggesting a lower level of lipid peroxidation and better membrane stability. In agreement, a previous study showed that SA could effectively reduce MDA content in guava fruits [38]. Our results reveal that exogenous SA treatment could play a crucial role in extending the shelf life of sand pear fruits during shelf life at room temperature by increasing antioxidant enzyme activity and promoting membrane stability. The combined action of different antioxidant enzymes might be a central mechanism involved in removing ROS elements and delaying fruit senescence in sand pear fruits. During the ripening and senescence of fruits, metabolic imbalances lead to an excessive accumulation of ROS, resulting in membrane lipid peroxidation and exacerbating the aging process [12,89,90]. This accumulation further accelerates senescence [10,91]. To counteract this, plants employ a comprehensive antioxidant defense system, comprising both enzymatic and non-enzymatic mechanisms, to mitigate ROS overaccumulation. Key enzymes, including SOD, POD, CAT, and APX, play pivotal roles in maintaining ROS levels within safe thresholds [11,86,92]. The activation of these antioxidant enzymes is vital for preserving redox balance through ROS reduction [93,94,95,96]. Previous studies have established the critical function of POD, CAT, and APX in extending fruit shelf life by minimizing the excessive hydrogen peroxide produced in fruit tissues [6,97,98,99]. SA significantly influences the modulation of these antioxidant enzymes, thereby directly affecting fruit senescence and longevity [100,101]. Our results demonstrate that SA treatment enhances the activity of SOD, POD, CAT, and APX, thereby improving the pear’s capacity to neutralize ROS and reduce oxidative stress. SA application has been shown to augment fruit quality and extend postharvest shelf life in lemons and peaches through the upregulation of these antioxidant enzymes [100,101,102].

SA can upregulate the expression of genes encoding antioxidant enzymes. It interacts with specific transcription factors or signaling molecules, leading to the activation of the promoter regions of genes responsible for the synthesis of POD, SOD, and CAT, thus activating these enzymes in the cell [100]. In this study, the expression of the *PpSOD1* and *PpAPX6* genes decreased during shelf life but increased with exogenous SA treatment, while the expression of *PpPOD1* and *PpCAT1* remained unaffected by the SA application. SA treatment has been documented to postpone fruit senescence through modulating both the antioxidant system’s gene expression and senescence-associated genes. This results in enhanced fruit antioxidant capabilities, the stabilization of ROS equilibrium, and a reduction in membrane lipid peroxidation and browning [1,100,103,104]. While SA treatment did not significantly alter the expression levels of *PpPOD1* and *PpCAT1* during room temperature shelf life, it increased the activities of POD and CAT. Notably, CAT activity in pear fruit played a minor role in H_2_O_2_ scavenging [105]. Thus, various genes responsible for antioxidant enzyme activities in pear fruit exhibit differential responses to enzyme activity enhancement [106]. GST is a pivotal enzyme ubiquitously present in living organisms, playing a crucial role in protecting plants from oxidative damage through the role of glutathione peroxidase [18]. In the present study, we observed a significant upregulation in the expression of *PpGST2* in sand pears induced by exogenous SA treatment throughout the shelf life at room temperature. The increase in *GST2* expression could promote the accumulation of GST, potentially leading to a reduction in hydrogen peroxide levels during the senescence process.

There are extensive interactions between plant hormones during fruit senescence [23]. As a typical climacteric fruit, postharvest ethylene production is the leading cause of fruit senescence in sand pears. The ethylene signaling pathway is crucial in plant growth, development, fruit ripening, and senescence [20]. Ethylene synthesis is mainly regulated by two key enzymes, ACS and ACO [25]. SA and ethylene often play an antagonistic role in fruit senescence [107]. In this study, the ethylene biosynthesis gene of sand pears, *PpACO2,* was dramatically down-regulated by exogenous SA application during shelf life at room temperature. In contrast to our finding, SA treatment promoted the expression of *PpACO2* in sand pear fruits [62], which could be due to different SA concentrations and sampling times after treatment. EIN3 is a crucial member of an important gene family in plants that plays a significant role in ethylene signaling [27]. A previous study showed that SA could down-regulate the expression of the *PpETR2* and *PpERF113* genes in pear fruit [63]. Similarly, exogenous SA treatment inhibited the expression of the *PpEIN3a* gene in sand pear fruit during shelf life at room temperature. These results suggest that SA inhibits ethylene synthesis and signal transduction to delay pear fruit senescence. The NPR gene plays a pivotal role in the SA signaling pathway [108]. Here, we found that SA spray could upregulate the expression of the *PpNPR-1* gene in sand pear fruit during shelf life at room temperature, suggesting that exogenous SA may promote the SA signal transduction pathway in sand pears.

Auxins play an important role in fruit growth, but act as inhibitors of ripening in both climacteric and non-climacteric fruits [54]. Tryptophan transaminase (TAR) is an essential enzyme in the IAA synthesis pathway. The activation of the *TAR* gene leads to the synthesis of IAA, which subsequently delays fruit senescence [66]. Melatonin (MT), a tryptophan derivative, exhibits anti-senescence and other biological functions in plants [108]. Silencing the *SlCOMT1* gene could accelerate tomato senescence [67]. We found that SA could upregulate the expression of *PpTAR2* and *PpCOMT*, suggesting that SA extends the shelf life of pear fruit by increasing the biosynthesis of IAA and MT. SA and ABA play opposite roles in fruit senescence [109]. ABA accelerates fruit ripening by stimulating endogenous ABA biosynthesis. A study showed that suppression of the *NCED* gene could effectively delay fruit ripening and senescence [63]. Additionally, the mutation of Allene oxide cyclase (AOC), a crucial enzyme in jasmonic acid synthesis, prolonged fruit shelf life [64]. Interestingly, SA downregulates *PpNCED1* and *PpAOC2* in pear fruit, leading to the inhibition of ABA and JA biosynthesis. Thus, SA treatment modulates hormone biosynthesis and metabolism, thereby extending pear fruit senescence.

Flesh firmness has an important effect on the shelf life of fruit [110]. Cell wall metabolism plays a critical role in fruit senescence, with PG, PME, and CEL being central to this process [111]. PG is involved in the depolymerization of pectin in the cell wall, leading to the softening of fruit tissue. PME modifies the pectin by demethylating it, which makes pectin more susceptible to degradation by PG and other cell wall-degrading enzymes. CEL contributes to the breakdown of cellulose, further facilitating cell wall disassembly during senescence. Together, these enzymes orchestrate the complex remodeling of the cell wall, which is a hallmark of fruit ripening and senescence, affecting texture, firmness, and overall fruit quality [112,113]. Treatments with MT and 1-MCP suppress the expression of *PG*, *PME*, and *CEL*, consequently delaying pear fruit softening [68]. In this study, the application of SA inhibited the expression of *PpPG1*, *PpPME2*, and *PpCEL3*, thereby maintaining fruit firmness. PGIPs are binding proteins located in the cell wall. It specifically binds to and inhibits the activity of the fungal endoglucanase polygalacturonidase [57,58]. Here, the expression of *PpPGIP1* was upregulated by SA treatment, suggesting that *PpPGIP1* could delay fruit senescence and protect pear fruits from fungal pathogens during shelf life at room temperature.

The sugar and acid content of fruits is a quantitative trait governed by multiple genes. Fruit bagging treatment, which reduces light intensity, has been shown to reduce the expression of the *SPS* gene, thereby inhibiting sucrose synthesis [70]. Conversely, overexpression of the *SUS* gene enhances the sucrose content and biomass yield in transgenic sugarcane [71]. In this study, SA spray upregulated the expression of *PpSPS1* and *PpSUS1*, consequently promoting sucrose accumulation during shelf life. The expression pattern of *PbSOT6* and *PbSPT20* was correlated with the sorbitol accumulation pattern in pear fruit. Additionally, exogenous sorbitol induced the expression of *PbSOT6* and *PbSPT20* [72]. The sugar transporter genes (*PbTMT2*, *PbTMT3*, and *PbTMT4*) are closely associated with sugar accumulation levels during the developmental and ripening stages of pear fruit [73]. In pear fruit, *PuSWEET15* is responsible for sucrose transport. Overexpression of this gene leads to an increase in sucrose content, whereas silencing reduces sucrose levels [74]. Here, SA upregulated the expression of *PpSOT1*, *PpTMT4*, and *PpSWEET15*, thereby enhancing sugar transport. In most pear varieties, the primary organic acids are malic acid and citric acid. 1-Methylcyclopropene (1-MCP) fumigation upregulated the expression of *cyNAD-MDH* and reduced cyNADP-ME, thus maintaining a higher abundance of malic acid in pear fruit during storage [75]. Consistent with previous research, SA treatment upregulated the expression of *PpcyNAD-MDH* and inhibited *PpcyNADP-ME* expression, thus increasing the malic acid level in pear fruit during shelf life.

Previous studies showed that fruit browning and membrane lipid peroxidation strongly correlated with antioxidant defense systems [104,114,115]. In this study, the antioxidant defense enzymes and their gene expression, genes involved in ethylene biosynthesis and signaling transduction, and other senescence-related genes showed strong positive to negative correlations. SA treatment inhibited the increase in PPO activity and MDA content in fruits. Correlation analysis showed that CAT and APX activity were negatively associated with PPO activity and MDA content, respectively. However, the correlation was not significant, suggesting that SA could better maintain CAT and APX activity, which was conducive to reducing the damage of excessive ROS on fruit cell membranes. Similar results were observed in cherries [104]. SOD activity was positively correlated with POD activity and negatively associated with CAT and APX activity. This suggests that the stable metabolism of ROS during fruit storage may be the result of the interaction between SOD, POD, APX, or CAT rather than a single antioxidant enzyme. The coordination work of different enzymes facilitates the rapid removal of accumulated reactive oxygen species and protects fruit cells from oxidative damage [104]. Sugar and acid metabolism in pear fruit involves a complex process, with plant hormones playing a crucial role in its regulation [116]. Applying IAA or ABA to pears enhanced sorbitol accumulation in fruits [116]. During ripening, MT increases the levels of soluble sugars, mainly sucrose and sorbitol [61]. We found a significant correlation between plant hormone synthesis and the expression of genes related to sugar and acid synthesis. These results suggest that the various traits influenced by SA collectively contribute to delayed senescence in sand pear fruits.

SA, as a natural and safe phenolic compound, demonstrates tremendous potential for mitigating postharvest fruit loss [117]. SA can serve as a suitable alternative to chemical agents in postharvest horticultural practices, thus enhancing food safety. Despite this, SA, similar to other postharvest treatments, may yield varied effects across different crops and environments [118]. Previous research indicates that the use of 2 mM SA can preserve the postharvest quality of various fruits, including tomatoes [119], peaches [120], strawberries [121], and sweet cherries [122], thereby extending their storage duration. In this study, the application of 2 mM SA increased the antioxidant capacity of pear fruit and extended their shelf life. Consistent with previous research, a concentration of 2 mM SA proves to be a rational choice for delaying the senescence process in pear fruit. Accumulating literature showed that previous studies only focused on the effects of SA spray on extending fruit senescence, and studies did not indicate whether the SA-treated fruits could be directly used for human consumption. Therefore, further studies are needed to investigate whether the SA-treated fruits are safe for direct human consumption.

In summary, exogenous SA treatment effectively maintained pear fruits’ coloration and quality during room temperature shelf life. This preservation was associated with the regulation of antioxidant enzyme activity and the expression of related genes. SA treatment counteracted the harmful effects of room temperature shelf life on antioxidant enzyme activity, reduced PPO activity, and decreased lipid peroxidation. Additionally, SA treatment regulates ethylene biosynthesis and signal transduction, and the expression of senescence-related genes. These findings contribute to a better understanding of the mechanisms underlying the beneficial effects of SA treatment on fruit color and quality preservation during shelf life.

## 4. Materials and Methods

### 4.1. Fruit Materials and Treatments

‘Whangkeumbae’ (*Pyrus pyrifolia* Nakai) fruit samples were collected from the experimental farm of Hebei Agricultural University (Baoding, Hebei, China) at 150 days after full bloom (DAFB). The selected fruits had a uniform size and were free from damage, pests, and diseases. After collection, the fruits were immediately and carefully transported to the laboratory.

The collected samples were randomly divided into two groups, each containing 15 fruits. The SA treatment concentration (2 mmol L^−1^) was based on previous studies [42,100]. Pear fruits sprayed with SA solution and distilled water for 10 min were set as the treatment and control groups, respectively. The ethylene production of the pear fruits peaked at 10 days after harvest [123]. There were three replications, each containing five fruits. After treatment, fruits were air-dried at room temperature, and mesocarp discs were prepared. The collected mesocarp discs were immediately frozen with liquid nitrogen and stored at −80 °C.

### 4.2. Fruit Appearance Observation

For SA treatment, sand pear fruits were sprayed with 2 mmol L^−1^ SA for 10 min, and the control (CK) group was sprayed with ddH_2_O. The CK and SA-treated fruits were kept at room temperature for 0 days, 5 days, and 10 days, respectively. SA-treated fruits and untreated fruits were photographed at the same time.

### 4.3. Extraction and Assay of PPO Activity

The PPO activity was determined using a previous method [124], with a slight modification. First, 1 g of frozen pulp sample was homogenized with 5 mL of extraction buffer and centrifuged at 12,000× *g* for 30 min at 4 °C. The supernatant was collected for the PPO activity assay. A reaction solution was prepared by mixing 4 mL of 50 mmol L^−1^ sodium acetate buffer (pH 5.5) and 1 mL of 50 mmol L^−1^ catechol solution. Then, 100 µL of enzyme extract was added to the reaction solution, and the absorbance was recorded at 420 nm using a UV spectrophotometer (UV-5500, Metash, Shanghai, China). PPO activity was calculated as U g^−1^. This experiment was repeated three times.

### 4.4. Determination of Malondialdehyde (MDA) Content

For MDA content analysis, the crude enzyme was extracted according to the method of Cao [124], with slight modifications. In brief, 1 g of frozen pear sample was homogenized with 5 mL of trichloroacetic acid solution (10%) and centrifuged at 10,000× *g* for 30 min at 4 °C. Then, 2 mL of the supernatant was mixed with 2 mL of thiobarbituric acid solution (0.67%). The mixture was boiled in a boiling water bath for 20 min, cooled to room temperature, and centrifuged at 10,000× *g* for 30 min at 4 °C. The absorbance was measured at 450 nm, 532 nm, and 600 nm using a UV spectrophotometer. MDA content was calculated as μmol g^−1^. This experiment was repeated three times.

### 4.5. Crude Enzyme Extraction and Antioxidant Enzymes Activity Assay

For crude enzyme extraction, 1 g of tissue was ground and homogenized with 10 mL of phosphate saline buffer (pH 7.8). Then, the mixture was transferred into a 15 mL tube and centrifuged at 12,000× *g* for 15 min at 4 °C. The supernatant was carefully collected and used to measure SOD, POD, and CAT activity [124,125]. SOD activity was measured according to a previous method and slightly modified [124,125,126]. Briefly, the reaction solution was prepared by mixing 2 mL of sodium dihydrogen phosphate buffer (50 mmol L^−1^, pH 7.8), 0.5 mL of methionine solution (104 mmol L^−1^), 1 mL of nitro-blue tetrazolium (NBT) solution (300 µmol L^−1^), 0.5 mL of ethylenediamine tetraacetic acid solution (80 mmol L^−1^), and 50 µL of riboflavin solution (320 µmol L^−1^). Subsequently, 0.5 mL of crude enzyme extract was added to the reaction solution. The mixture was shaken and immediately placed under a 4000 lux fluorescent lamp for 15 min, and the reaction was terminated immediately by putting it in the dark. In addition, the control group was treated in the same way as above, except that no enzyme extract was added. Then, the absorbance of the mixture was measured at 560 nm. The SOD activity was calculated as U g^−1^. POD activity was determined according to the method described previously with slight modifications [124,125]. Briefly, a reaction system consists of 100 mL of sodium dihydrogen phosphate buffer (50 mmol L^−1^, pH 7.8), 0.038 mL of guaiacol solution (50 mmol L^−1^) and 0.056 mL of H_2_O_2_ (30%) solution. The reaction solution (3 mL) was combined with 0.1 mL of crude enzyme extract. Then, the absorbance was measured at 470 nm using a UV spectrophotometer. POD activity was calculated as U g^−1^. CAT activity was determined according to previous methods [124,125,126] with slight modifications. In brief, 2 mL of 50 mmol L^−1^ sodium dihydrogen phosphate buffer (pH 7.8), 1 mL of 45 mmol L^−1^ H_2_O_2_ solution, and 0.5 mL of supernatant were mixed and immediately measured for absorbance at 240 nm using a UV spectrophotometer. CAT activity was given as U g^−1^. All enzyme assay experiments were performed with three replications.

### 4.6. Crude Enzyme Extraction and Ascorbate Peroxidase (APX) Activity Assay

The APX activity was determined according to the method described by Cao [125] and Zhu [127], with a slight modification. About 1 g of fruit tissue was homogenized with 5 mL of extraction buffer containing 0.1 mmol L^−1^ ethylenediamine tetraacetic acid (EDTA), 1 mmol L^−1^ ascorbic acid, and 2% polyvinylpyrrolidone (PVPP) and centrifuged at 12,000× *g* for 30 min at 4 °C. Then, the supernatant was collected and used to determine APX activity. About 0.1 mL of enzyme extract was mixed with 2.6 mL of a reaction system consisting of 0.1 mmol L^−1^ EDTA, 0.5 mmol L^−1^ ascorbic acid, and 2 mmol L^−1^ H_2_O_2_ solution. The absorbance was measured at 290 nm. The APX activity was calculated as U g^−1^. This experiment was repeated three times.

### 4.7. Quantitative Real-Time PCR (qRT-PCR) Expression Analysis

Total RNA was extracted using the RNAprep Pure Plant Plus Kit (Polysaccharide and Polyphenolic-rich) RNAprep Pure (Tian Gen, Beijing, China), according to the instructions. The quality and quantity of the RNA were checked using NanoDrop spectrophotometry (Thermo, Shanghai, China). Then, cDNA was synthesized from 2 µg of RNA using a FastQuant RT Kit (with gDNase) (Tian Gen, Beijing, China). Real-time qRT-PCR analysis was performed using the Magic SYBR mix (Cwbio, Beijing, China) on the Mastercycler ep realplex 4 assay system (Eppendorf AG, Hamburg, Germany). All the qRT-PCR reactions were performed in the 20 μL total sample volume, including 10 μL of 2× SYBR Mixture (Cwbio, Beijing, China), 0.4 μL of each primer (10 mmol L^−1^), 7.2 μL of nuclease-free water, and 2 μL of diluted cDNA. The PCR procedure was as follows: 30 s template predenaturation at 95 °C, 5 s template denaturation at 95 °C, 30 s primer annealing at 60 °C, and 10 s primer extension at 72 °C for 42 cycles, followed by the melting curve analysis. The *PpUbqutin* gene was used as an internal control to normalize gene expression. Data were analyzed using the 2^−ΔΔCT^ method [128]. Gene-specific primers used for expression analysis are listed in Table 1.

### 4.8. Statistical Analysis

Analysis of variance (ANOVA) was employed to analyze the data using Statistical Package for the Social Sciences (SPSS) software version 22.0 (IBM Co., San Francisco, CA, USA). All values are shown as the mean ± SE (n = 3). The confidence levels of the statistically significant differences were analyzed using Duncan’s test at * *p* < 0.05, ** *p* < 0.01, and *** *p* < 0.001. GraphPad Prim 8.0 (San Diego, CA, USA) was used to draw graphs, and Origin 2021 software (Origin Lab, Inc., Northampton, MA, USA) was used for correlation analysis.

## 5. Conclusions

This study demonstrates the role of exogenous SA spray in regulating fruit senescence during shelf life at room temperature in sand pears. The results showed that the samples treated with SA had higher antioxidant capacity, as shown by increased SOD and APX activity and an up-regulation of *PpSOD1*, *PpPOD1*, *PpCAT1*, *PpAPX6*, and *PpGST2*, which might account for the lower MDA content. In addition, SA treatment decreased the expression of the ethylene biosynthesis gene *PpACO2*, the ethylene signal transduction-related gene *PpEIN3a*, the ABA synthesis-related gene *PpNCED1,* and the JA synthesis-related gene *PpAOC2*. Furthermore, SA increased the expression of the SA signal transduction-related gene *PpNPR1*, the IAA synthesis-related gene *PpTAR2*, and the MT synthesis-related gene *PpCOMT1*. Additionally, SA spray down-regulated cell wall metabolism-related genes (*PpPG1*, *PpPME2*, and *PpCEL3*) and up-regulated sugar and acid metabolism-related genes (*PpSPS1*, *PpSUS1*, *PpSOT1*, *PpTMT4*, *PpSWEET15*, and *PpcyNAD-MDH*). Overall, our results suggest that postharvest SA treatment can be used as an effective strategy to increase the shelf life of sand pear fruit at room temperature and maintain fruit quality for extended periods. Moreover, previous studies only focused on understanding the effects of SA application on fruit physiology, and no research has been done on the effects of consuming SA-treated fruits on human health. Therefore, more research is required to determine the possible health effects of consuming fruits treated with SA.

## Figures and Tables

**Table 1:** Gene-specific primers used for expression analyses.

Gene Name	Forward Primer (5′-3′)	Reverse Primer (5′-3′)
*PpPPO1*	TCCCTACTCACAAAGCCCAAG	GACCTCCAAGACCAAGAAGCA
*PpSOD1*	TTCCCTACGACTATGGGGCT	GTTGGTGACGTAAGCCTGGT
*PpPOD1*	AAGGCATGCATGTGGTCAGT	CGACATATCCACCATGCCCA
*PpCAT1*	GCAACTACCCGGAGTGGAAA	TACCAAACGTCCAACTGGCT
*PpAPX6*	TCATTGGGACACCCAACAGC	GACATGCCGGGAATTGAAGC
*PpGST2*	CTTATCTTTTGGATGGATAGCATTC	CGTGTTTGGTTCATGTATATATATTA
*PpACO2*	ATGGAGAACTTCCCAGTTATCAACT	TCAAGCAGTAGTTTTAACTGGACCC
*PpEIN3a*	GGAGTTGATGATGGGCAGAAAATG	GGTTCAGACATGTTGATGTTGCAT
*PpNCED1*	TCGTCTACCACAACTGCCAC	TCGTAGCTAAGCGCGAAGAG
*PpAOC2*	GGACACGTATCTGGCTGTGA	AAGCTCCTCAGGCAAATCCT
*PpNPR-1*	TTCTCACTAAAGGAGCTCGTGCGT	GCAACTCCATGTGCAGATCATCAG
*PpTAR2*	CCTTGCTGGTTTTTGGAGCC	GGACTTCAAGCAGTCCGTCA
*PpCOMT1*	GGCCTCACATCCATCGTTGA	GCGCAGCATAGCAGTTCTTC
*PpPG1*	ATGGCTTTAAAAACACAGTTGTTGT	ACCTTCAATGGTGTCCATGTATG
*PpPME2*	ACCATCCTTGTTCGTCCTAGACA	GTCTCCCCAGGTAGTTCTTGTGC
*PpCEL3*	GCTCCACTGCCTAAAGTTGCTC	GGCCCCAAATAGGACCGTAAAG
*PpPGIP1*	CTTCGTCTAGACCGCAATAAGCTCA	ATTGCTGGCCAAATCTGCAGTTGTG
*PpSPS1*	ACTCATGAGAATTCAGGCTCTC	GTCGTCTGGAAAGACATGTTC
*PpSUS1*	ATGGAGAATCGCCGTAAGTTC	AAGGTCCATTTTTGAGCTGCTG
*PpSOT1*	TCCAATTACGTGGGTTTACAGTTC	TGGAACTTGCCAAACAAGACC
*PpTMT4*	GGGTTCTTCTTTCGAACTTGG	TCTGGTGGGAAAGATTTCTGC
*PpSWEET15*	TTTATGCACCAAAGGAAGCTAGG	ACACAAAACCCAGAACGTTTGG
*PpcyNAD-MDH*	TGCTGAGGCTTTGAATGGTG	TTTCCAGTGCAGAAGCTTGG
*PpcyNADP-ME*	GTGGGAAAACAATTCACGAAGG	ACTTTAGCAGCAATGTTGGCTG
*PpUBI*	TGCAGATCTTCGTGAAAACCCTAAC	CATAGCACTTGCGGCAGATCATCT

## Data Availability

Data are contained within the article.
